# Association between Dietary Habits, Shift Work, and the Metabolic Syndrome: the Korea Nurses’ Health Study

**DOI:** 10.3390/ijerph17207697

**Published:** 2020-10-21

**Authors:** Heeja Jung, Hyunju Dan, Yanghee Pang, Bohye Kim, Hyunseon Jeong, Jung Eun Lee, Oksoo Kim

**Affiliations:** 1 College of Nursing, Konyang University, Daejeon 35365, Korea; jhj1215@konyang.ac.kr; 2 College of Nursing, Ewha Womans University, Seoul 03760, Korea; hidan@hanmail.net (H.D.); anais0220@hanmail.net (Y.P.); bohyekim516@naver.com (B.K.); idadoshi@naver.com (H.J.); 3 Department of Food and Nutrition, College of Human Ecology, Seoul National University, Seoul 08826, Korea; jungelee@snu.ac.kr

**Keywords:** metabolic syndrome, nurses, eating habit, shift work

## Abstract

Metabolic syndrome (MetS) is an important public health problem, and unhealthy dietary habits and shift work are considered major factors that increase the prevalence of MetS. The purpose of this study was to examine whether dietary habits, alcohol drinking, and shift-working were associated with development of MetS in shift-working female nurses. This study analyzed cross-sectional survey data from the Korea Nurses’ Health Study (KNHS). Of the 1638 nurses, 403 participants were selected based on the propensity score matching method (PSM). These participants had either no or more than three MetS determinant factors. Analysis was conducted by using multivariable logistic regression to confirm the factors influencing MetS. The prevalence of MetS in this group (1638 participants) was 5.6% (92 participants). Consumption of over 50% of daily calorie intake after 7 p.m., consumption of carbonated drinks, family history of diabetes, and non-shift work were significant factors influencing MetS. Nurses are one of the at-risk groups for unhealthy dietary habits due to the nature of their work. Therefore, nurse managers should include regular dietary education for nurses and continue their policy efforts to resolve health problems that may arise in connection with nurses’ work.

## 1. Introduction

Metabolic syndrome (MetS) is an important public health problem. MetS is associated with an increased incidence of and death due to chronic diseases such as cardiovascular disease [1]. MetS is defined as a disease in which three or more of the five symptoms, abdominal obesity, high blood pressure, high fasting plasma glucose, hypertriglyceridemia, and low level of high-density lipoprotein (HDL) cholesterol, are present simultaneously [2]. In the US, MetS prevalence in 2014 was 23% [3], while the prevalence in Korea as of 2017 was 28.1% for men and 18.7% for women.

Major risk factors for MetS among the general adult population include age [4,5], higher BMI [5] family history [6,7], physical inactivity [4,8], and unhealthy dietary habits [9]. In addition, factors including eating pattern [10], coffee consumption [11,12], alcohol consumption [13], and shift work [14] are influencing factors for MetS. This study focuses on investigating the association of MetS with factors like work characteristics of nurses and lifestyles of childbearing women among various influencing factors of MetS and considered these factors as main variables.

Fast eating speed is relevant to weight increase and the risk of MetS development. According to a systematic literature review, fast eating speed is associated with increase of body mass index (BMI) and obesity [15]. Among Chinese adults aged between 18 and 65 years, fast eating is significantly associated with high incidence of MetS and is related to elevated blood pressure and abdominal obesity among the components of MetS [16]. A study on Korean hospital nurses’ dietary habits reported that time allocated for a meal while on duty was 16–28 min on average; this differed depending on work shift. Mealtime for the day shift working nurses was the shortest [10].

Soft drink, such as sugar-sweetened or artificially sweetened beverage, intake is known to be a factor increasing MetS risk [17,18]. Furthermore, frequent sugar-sweetened drink consumption is associated with a higher risk of type 2 diabetes [19]. In Korea, the 10–20 year old group consumes the most sugar, and sugar-sweetened beverages are the major source of sugar intake from processed foods, the largest proportion of which was carbonated drink consumption [20]. Pae et al. [21] reported the association between frequent carbonated drink consumption and women’s obesity. However, studies on the relationship between carbonated drink consumption and MetS incidence are infrequent.

Although the results on the association between coffee consumption and MetS in a general adult group are not consistent across studies [22,23], coffee consumption is reported to reduce MetS prevalence in women from South Korea [11,12]. In particular, according to a study that analyzed the data from the Korean National Health and Nutrition Examination Survey [24], consumption of brewed coffee is rapidly increasing in South Korean young women, increasing the need for investigation on the relationship between black coffee consumption and MetS.

The relationship between alcohol intake and MetS incidence has been reported based on the amount of alcohol intake and sex. A daily alcohol consumption of 30 g by a Polish male adult was associated with high incidence of MetS, whereas a daily consumption of 10.1–15.0 g by a Polish female adult was associated only with abnormal blood sugar level among the components of MetS [13]. In South Korea, MetS incidence was lower in male and female adults whose daily consumption was 0.1–5.0 g (very-light drinker) than in non-drinking male and female adults, whereas heavy drinking adults with a daily alcohol consumption of 30.0 g or greater appeared to have no significant association with MetS occurrence [25].

A first-degree family history of diabetes is a risk factor for MetS. Based on a study conducted on Chinese adults, MetS incidence in first-degree relatives (FDRs) was reportedly higher than in non-FDRs [6,26]. In South Korea, MetS prevalence in adults aged between 25 and 44 years with a family history of diabetes was 21.3%, which is significantly higher than that of adults without a family history of diabetes (12.1%). Furthermore, MetS occurrence was higher in adults with family history of large waist circumference, high triglyceride level, and high blood pressure [27]. Familial combined hyperlipidemia appeared along with MetS (coexistence or comorbidity) in many cases [28,29], and the presence of family history of hypertension appeared to significantly increase the incidence of MetS [30].

Despite the insufficient evidence on the relationship between shift work and prevalence of MetS [31], sleep deprivation due to shift work appears to increase the risk of visceral obesity, a critical diagnostic criterion of MetS [32,33,34]. In addition, shift-working nurses showed wider day-to-day caloric intake variability than non-shift-working nurses, and such eating behavior was related to an increase in waist circumference and body mass index [35]. Nurses working in shifts were reported to consume more high-calorie fast food and snacks than daytime workers [36], and such eating behavior may cause obesity and ultimately increase MetS risk. Fragmented studies on the relationship between shift work and MetS have been conducted; few studies confirmed that poor dietary habits are an important trait of shift workers.

In Korea, nurses are among the main occupational group who work shifts. We assumed that the changes in dietary habits of young women, as well as shift work, would affect the incidence of MetS. Therefore, this study was intended to analyze the data of the Korea Nurses’ Health Study, a large-scale prospective cohort study, to examine whether dietary factors, alcohol drinking and shift-working were associated with prevalence of MetS among female nurses.

## 2. Materials and Methods

### 2.1. Study Design and Participants

This study analyzed cross-sectional survey data from the Korea Nurses’ Health Study (KNHS), a large-scale prospective cohort study that started in 2013. The primary goal of the KNHS is to investigate the effect of occupational and lifestyle characteristics of female nurses of childbearing age on their health. For KNHS data collecting, 20,613 participants answered the first phase 1 website survey [37]. Participants of the phase 1 survey were invited through a text message to continue with subsequent online surveys that were taken via a website and mobile access. For phase 1 of the KNHS, survey modules 1–4 were conducted from 2013 to 2015; subsequent survey modules 5–7 were conducted from 2016 to 2019 as phase 2. Phase 3 started in 2019 and is currently in progress. In conjunction with the module 5 survey during 2016–2017, we collected voluntary blood samples from 1699 phase 1 survey participants from 12 hospitals.

The inclusion criteria of the study were nurses aged between 20 and 45 years working at a hospital at the start of the KNHS survey in 2013. There were no exclusion criteria for the survey. For blood collection, pregnant women and women in menopause were excluded as items like AMH were included in the blood test. The survey was conducted online, and all the questions needed to be answered before moving to the next page, ensuring no missing answers.

### 2.2. Measure

#### 2.2.1. Metabolic Syndrome (MetS)

MetS is defined as the presence of three or more of the following five risk factors established by the National Cholesterol Education Program-Adult Treatment panel III [38] based on abdominal obesity criteria for Asian-specific from the International Diabetes Foundation [39]: (1) abdominal obesity (WC ≥80 cm in women); (2) hypertriglyceridemia (triglycerides, TG ≥150 mg/dL); (3) low level of high-density lipoprotein, HDL cholesterol (<50 mg/dL in women); (4) high blood pressure (systolic BP ≥130 mmHg and/or diastolic BP ≥85 mmHg); (5) high fasting plasma glucose (≥100 mg/dL). In this study, participants with more than 3 of the above-mentioned risk factors of metabolic syndrome were classified into the MetS group and participants with 0 risk factor were classified as the normal group.

#### 2.2.2. Biochemical Evaluations

Informational materials including preparations such as fasting for 8 h before the blood test were delivered to the participants with the help of the nursing department at each hospital. Medical technologists drew blood samples through a venipuncture of the median antecubital vein through a vacuum system after 8 h of fasting. The blood drawn was stored, transported to a certified laboratory (Green Cross LabCell, Yongin, Korea), and TG, HDL-cholesterol, and plasma glucose were analyzed with an autoanalyzer (Cobas 8000, Roche Diagnostics, Mannheim, Germany).

#### 2.2.3. Anthropometric Measurements

Two well-trained registered nurses performed anthropometric measurements of the participants wearing light clothing. WC was measured to the nearest 0.1 cm from the narrowest point between the lower borders of the rib cage and the iliac crest at the end of normal expiration. Weight and height were based on self-report data from the fifth survey of the KNHS 2. The BMI was calculated by dividing weight (kg) by height squared (m^2^) [40].

#### 2.2.4. Blood Pressure Measurement

Nurses participating in blood tests were asked to measure and record blood pressure before the test. Blood pressure was measured using an automatic sphygmomanometer. Before measurement, nurses were told to stop nursing tasks, rest for 10 min, and measure blood pressure in a seated position.

#### 2.2.5. Additional Variables

A question on whether the participant worked in shifts was included to reflect the characteristics of their occupation. Shift work was defined as “working in day shift (7 a.m. to 3 p.m.), evening shift (3 p.m. to 11 p.m.), and/or night shift (11 p.m. to 7 a.m.) consecutively.”

To assess the relationship between MetS and habitual eating behavior, we asked the participants to estimate their eating speed and to determine whether more than 50% of their daily calorie intake occurred after 7 p.m. To assess this, we asked ‘Do you take over 50% of daily calorie intake after 7 p.m. ?’; the participants answered ‘yes’, or ‘no’. Eating speed was categorized as either ‘less than 10 min’, ’10–15 min’, and ‘over 15 min’.

We calculated daily alcohol consumption by asking the average drinking frequency over the past year, the type of alcohol consumed, and the consumption amount per occasion. We categorized the participants into ‘Non-drink,’ ‘<1 cup/day,’ and ‘1≤ cups/day’ groups. Consumption of black coffee, including one cup of Americano, espresso, and instant black coffee mix over the past year was also ascertained. Again, the participants were categorized into ‘Non-drink,’ ‘<1 cup/day,’ and ‘1≤ cups/day’ groups. Regarding the consumption of carbonated drinks over the past year, the frequency and portion sizes were ascertained from the food frequency questionnaire [41]. The participant groupings were: ‘Non-drink,’ ‘<1 cup/day,’ and ‘1≤ cup/day’. Carbonated drinks included cola, cider, and fruit-flavored soda. Participants were asked if either of their parents had been diagnosed with diabetes, hypertension, and hyperlipidemia to confirm family history.

### 2.3. Ethical Considerations

The KNHS was conducted with approval from the Institutional Review Board (Approval No. 117-4) at Ewha Womans University, Seoul. The participants confirmed the research purpose and confidentiality and agreed to the informed consent before participating in the study.

### 2.4. Statistical Analysis

We employed the Statistical Package for Social Sciences (SPSS) Version 24 (SPSS Inc, Chicago, IL, USA) for the data analysis. We used the propensity score matching method (PSM) to match the MetS group and the normal group and to reduce the bias of skewed samples between the two groups. The PSM is used to balance the distribution of covariates between two observed groups to reduce selection bias [42]. We reviewed previous studies [43,44], conducted the basic analysis and conducted nearest neighbor matching with caliper 0.2. The four general characteristics variables used were age, marital status, level of education, and salary. As a result, five participants with MetS who did not meet PSM conditions were excluded and 403 participants were selected for the final analysis: 87 participants in the MetS group and 316 participants in the normal group.

After the PSM, the frequency and percentage were calculated, and independent t-test and chi-square analysis was performed to confirm the difference between the two groups. Spearman’s correlation was also performed to confirm the relationships among the variables. Last, we performed multivariable logistic regression to confirm the factors influencing MetS, and the results were illustrated using the odds ratios (OR) and 95% confidence intervals (CIs). In this study, a p-value of less than 0.05 was considered statistically significant.

Eating speed was measured in three categories: ‘less than 10 min,’ ‘10–15 min,’ and ‘more than 15 min.’ The frequency and intake amounts of alcohol and coffee were measured, converted into cups/day, and categorized as ‘none,’ ‘<1 cup/day,’ and ‘1 or more cups/day,’ respectively. The measured frequency and intake amount of carbonated drinks were converted into servings/day and categorized as ‘none,’ ‘<1 serving/day,’ and ‘1 or more servings/day’ for analysis.

## 3. Results

Of the 1638 participants, 5.6% (92 participants) were confirmed to have MetS. Frequency and distribution of the final 403 research participants according to the key variables and the chi-square analysis results between the MetS and normal groups are illustrated in Table 1.

Fifth-four percent of the participants in the MetS group reported eating speeds of less than 10 min per meal, while 45.1% of normal group participants reported 10–15 min (χ^2^ = 12.212, *p* = 0.002). More participants in the MetS group than the normal group answered ‘yes’ to consuming more than 50% of daily calorie intake after 7 p.m. (χ^2^ = 13.464, *p* < 0.001). The two groups did not show any difference in terms of alcohol and black coffee consumption. However, the group with MetS consumed more carbonated drinks (χ^2^ = 15.002, *p* = 0.002). MetS group participants also reported a higher frequency of diabetes, hypertension, and hyperlipidemia in their family histories (χ^2^ = 14.810, *p* < 0.001; χ^2^ = 7.545, *p* = 0.006; χ^2^ = 6.484, *p* = 0.011, respectively). 

Table 2 presents the differences in MetS risk factors and BMI between the two groups. In particular, the triglycerides level differed significantly between the two groups: the MetS group averaged 178.07 ± 88.852 mg/dL and the normal group averaged 71.81 ± 23.879 mg/dL. The average BMI of the former group was categorized as overweight, 26.77 ± 3.267 kg/m^2^, while the average of the latter group was categorized as normal, 20.64 ± 1.904 kg/m^2^.

Correlation analysis among the variables revealed the relationships of eating speed (r_s_ = −0.150, *p* = 0.002), consuming more than 50% of daily calorie intake after 7 in the evening (r_s_ = 0.183, *p* < 0.001), carbonated drink consumption (r_s_ = 0.111, *p* = 0.026), family history of diabetes (r_s_ = 0.192, *p* < 0.001), family history of hypertension (r_s_ = 0.137, *p* = 0.006), and family history of hyperlipidemia (r_s_ = 0.127, *p* = 0.001) with MetS (Table 3).

Table 4 shows the result of multivariate logistic regression analysis performed to confirm the predictors of MetS. Factors influencing MetS were consuming over 50% of daily calorie intake after 7 p.m., consumption of carbonated drinks, family history of diabetes, and shift work. We found that the prevalence of MetS increased among participants who consumed more than 50% of daily calorie intake after 7 p.m. (OR = 2.681; 95%CI = 1.522–4.724). Participants with a family history of diabetes were approximately twice as likely to have MetS (OR = 2.077; 95% CI = 1.141–3.7784). The group of participants who consumed more than 1 serving of carbonated drinks per day on average had a 6.3 times higher MetS prevalence compared to non-drinkers (OR = 6.326; 95% CI = 1.908–20.971). Last, the group of participants who did not work on shifts were 1.76 times more likely to have MetS than the participants who worked on shifts (OR = 1.757; 95% CI = 1.022–3.021).

## 4. Discussion

The prevalence of MetS among Korean female nurses aged 24–48 years was calculated in this study to be 5.6%. This is much lower than the results of prior studies that reported an 18.7% MetS prevalence among Korean women over age 19 [45] and a 38.7% prevalence among female nurses aged 38–50 [46]. This difference in prevalence from our study is probably due to age difference. In our study, women of childbearing age, are younger than the subjects of the two preceding studies. A previous study that analyzed Korea National Health and Nutrition Survey data reported a 6.7% prevalence of MetS among Korean women aged 19–39 years [47].

Late night eating habits are related to MetS [48].The present study supported this conclusion by showing that consumption of calories late at night was a risk factor of MetS occurrence. In a preceding study on middle-aged adults without obesity and diabetes, adults consuming more than 48% of daily calories at dinner were reported to have a 1.5 times higher risk of developing MetS [49]. When the calorie from dinner and late-night snack is less than 1/2 of the recommended daily calorie intake, the risk of abdominal obesity decreases [50]. A study that applied the weight-loss program to overweight or obese women of childbearing age also revealed that consuming more calories at lunch than dinner was effective in weight loss, BMI reduction, and insulin resistance improvement [51]. The results from this study and preceding studies [49,50,51] confirm the need to educate women of childbearing age to ensure that calories are properly distributed and consumed at breakfast and lunch. Further studies are needed to elucidate how circadian rhythm and eating time play roles in the development of metabolic disorders in young women.

People with higher calorie intake over dinner tend to skip breakfast [49]. A prior study on the relationship between MetS and eating behavior of male and female adults reported that late dinner eating behavior did not raise the MetS risk on its own, but late dinner eating behavior together with skipping breakfast increased MetS risk [52]. These previous studies may suggest the evidence that breakfast and dinner eating behaviors are closely related. This study did not cover eating behavior other than dinner; a separate study that includes other eating behaviors, such as eating breakfast, shall be performed later.

In this study, consumption of soft drinks was a factor of increasing MetS risk. Most soft drinks, excluding zero-calorie products, usually contain 1–12% of sugar, and the over-consumption of sugar may cause adverse health problems such as obesity, diabetes, and fatty liver [53]. In a study of male and female adults aged 25 and older, increase in soft drink consumption increases waist circumference, increasing the risk of abdominal obesity [54]. In contrast, replacement of soft drinks with whole milk or juice decreased waist circumference [54]. Young Korean adults have a high rate of sugar consumption through beverages and a high tendency to consume soft drinks, highlighting the importance of managing beverage intake for young people [55,56]. Therefore, it is necessary to manage and educate young adults, especially women, about high intake of beverages with low calorie or sugar content. Although we were not able to examine total sugar or added sugar consumption in relation to MetS, our study warrants further investigation on the association between sugar consumption and metabolic disorders in Korean adults.

Increase in alcohol consumption has been shown to be a risk factor of MetS [57], but it did not have a significant impact on occurrence of MetS in the present study. This lack of significance is attributed to the inclusion of only female nurses, who tend to consume less alcohol than adults in general. In addition, although increase in coffee consumption could lower the risk of MetS [22], there was there was no significant relationship between coffee consumption and MetS occurrence in this study. Therefore, it is necessary to confirm this relationship in the future.

In this study, we identified that the subjects with a family history of diabetes were at over 2 times higher risk of MetS. This supports the finding of the prior study that a family history of diabetes is a predicting risk factor of MetS [26,27,58]. Adults aged 45 and younger may neglect life habit management, but their MetS incident risk is relatively low. However, those with a family history of diabetes need to adopt preventative life habits including diet management and increased physical activity to lower MetS incidence risk [27]. To this end, a workplace healthcare program offering preventative life habit improvement mediation to those having a high risk of MetS needs to be actively pursued.

Considering that young nurses mostly work on shifts, we confirmed the relationship between shift work and MetS while controlling for subjects’ age. In this study, daytime working nurses had a higher MetS risk than nurses working in shifts, which is different from the results of the prior studies [34,59]. The critical indicators of MetS of low HDL-cholesterol, and increased waist circumference (WC), could be related to lower physical activity [60]. We did not measure physical activity-related variables in this study. However, we speculated that the shift work nurses taking care of the patients would have a higher level of physical activity than non-shift work nurses with managerial roles. A study comparing the physical activity levels of hospital workers did not show a significant difference in leisure-time physical activity between the shift workers and non-shift workers. However, the shift workers were less sedentary and more physically active at work [61]. We can infer that the physical activity in the working environment may impact the relationship between shift work and MetS prevalence.

Therefore, it is necessary to measure physical activity level according to shift work and confirm its impact on critical indicators of MetS. Also, there is need to analyze physical activity in daily life through a follow-up study in addition to that in the work environment to confirm the impact on MetS.

The most critical method of MetS management is to mitigate the fundamental risk factors, including obesity, physical inactivity, and unhealthy dietary habits, through consistent lifestyle change [2]. Hence, if nurses correct their unhealthy dietary habits such as consuming more than 50% of daily calories after 7 p.m. and frequent consumption of carbonated drinks, MetS incidence should be reduced.

The limitations of this study are that the study did not confirm a causal relationship between risk factors and MetS occurrence. This is the product of the cross-sectional design of the study. Also, the study did not consider the influence of physical activity on MetS. However, the study has suggested factors associated with MetS among nurses and subsequently validated the necessity of eating behavior management for young working women and preventative life habit mediation for the subjects with diabetes family history.

## 5. Conclusions

Nurses usually do not have regular mealtimes while on duty; therefore, their dietary habits tend to include consumption of unhealthy drinks while working or large quantities of food at once after work. Therefore, nurse managers should include dietary education as job training for nurses and continue their policy efforts to resolve health problems that may arise in connection with nurses’ work. Also, we identified non-shift work as one of the important influencing factors of MetS; however, this result does not account for physical activity at work or during leisure-time. This indicates the need for further investigation considering variables related to activity level of shift workers and non-shift workers.

## Figures and Tables

**Table 1:** General characteristics of participants (N = 403).

Variables	Category	Total	Non-MetS	MetS	*x^2^*	*p*
		N (%)				
		403	316	87		
Meal speed (minutes)	15≤	95(23.6)	79 (25.0)	16 (18.4)	12.212	0.002 **
	10–15	155 (38.5)	131 (41.5)	24 (27.6)		
	<10	153 (38.0)	106 (33.5)	47 (54.0)		
Consuming more than 50% of calories per day after 7 p.m.	No	181 (44.9)	157 (49.7)	24 (27.6)	13.464	<0.001 ***
	Yes	222 (55.1)	159 (50.3)	63 (72.4)		
Amount of alcohol consumption (cups/day)	Non-drink	97 (24.1)	76 (24.1)	21 (24.1)	0.019	0.991
	<1	284 (70.5)	223 (70.6)	61 (70.1)		
	1≤	22 (5.5)	17 (5.4)	5 (5.7)		
Black coffee consumption (cups/day)	Non-drink	58 (14.4)	48 (15.2)	10 (11.5)	2.505	0.286
	<1	166 (41.2)	134 (42.4)	32 (36.8)		
	1≤	179 (44.4)	134 (42.4)	45 (51.7)		
Soft drink consumption (serving/day) (carbonated drink)	Non- drink	118 (29.3)	97 (30.7)	21 (42.1)	15.002	0.002 **
	<1	268 (66.5)	212 (67.1)	56 (64.4)		
	1≤	17 (4.2)	7 (2.2)	10 (11.5)		
Family history of Diabetes	No	308 (76.4)	255 (80.7)	53 (60.9)	14.810	<0.001 ***
	Yes	95 (23.6)	61 (19.3)	34 (39.1)		
Family history of Hypertension	No	182 (45.2)	154 (48.7)	28 (32.2)	7.545	0.006 **
	Yes	221 (54.8)	162 (51.3)	59 (67.8)		
Family history of Hyperlipidemia	No	281 (69.7)	230 (72.8)	51 (58.6)	6.484	0.011*
	Yes	122 (30.3)	86 (27.2)	36 (41.4)		
Shift work	No	162 (40.2)	122 (38.6)	40 (46.0)	1.541	0.214
	Yes	241 (59.8)	194 (61.4)	47 (54.0)		

* *p* < 0.05, ** *p* < 0.01, *** *p* < 0.001.

**Table 2:** Differences in Risk Factors for Metabolic Syndrome and Body Mass Index.

	Total	Non-MetS	MetS	t	*p*
Waist Circumference	72.57 ± 9.847	68.56 ± 5.185	87.14 ± 9.007	−18.414	<0.001 ***
Triglycerides	94.75 ± 63.654	71.81 ± 23.879	178.07 ± 88.852	−11.046	<0.001 ***
HDL-Cholesterol	65.22 ± 15.746	70.45 ± 12.864	46.23 ± 9.351	19.591	<0.001 ***
Systolic Blood Pressure	113.08 ± 9.603	110.82 ± 7.832	121.28 ± 10.937	−8.347	<0.001 ***
Diastolic Blood Pressure	71.67 ± 8.510	70.09 ± 7.623	77.38 ± 9.143	−6.809	<0.001 ***
Fasting Glucose	91.98 ± 12.219	88.39 ± 6.794	105.00 ± 17.607	−8.623	<0.001 ***
Body Mass Index	21.95 ± 3.392	20.64 ± 1.904	26.77 ± 3.267	−16.741	<0.001 ***

*** *p* < 0.001

**Table 3:** Correlations.

	1	2	3	4	5	6	7	8	9	10
1. Metabolic syndrome	1									
2. Meal speed	−0.150 **	1								
3. Consuming more than 50% of calories per day after 7 p.m.	0.183 **	−0.148 **	1							
4. Alcohol consumption	0.002	−0.024	0.120 *	1						
5. Black coffee consumption	0.078	−0.106 *	−0.014	0.114 *	1					
6. Soft drink consumption	0.111 *	−0.068	0.152 **	0.040	−0.032	1				
7. Family history of Diabetes	0.192 **	−0.132 **	−0.016	0.006	0.028	−0.020	1			
8. Family history of Hypertension	0.137 **	−0.070	−0.118 *	−0.080	0.040	0.107 *	0.304 **	1		
9. Family history of Hyperlipidemia	0.127 *	−0.058	−0.089	0.036	0.033	0.026	0.207 **	0.305 **	1	
10. Shift work	0.062	0.080	−0.053	−0.011	0.092	−0.134 **	−0.026	0.042	−0.022	1

* *p* < 0.05, ** *p* < 0.01.

**Table 4:** Result of the multivariate logistic regression analysis.

Variables	OR	95%CI	*p*
Meal speed			
15≤	1.00		
10–15	0.731	0.347–1.543	0.412
<10	1.671		0.149
Consuming more than 50% of calories per day after 7 p.m.			
No	1.00		
Yes	2.681 **	1.522–4.724	0.001
Amount of alcohol consumption (cups/day)			
Non-drink	1.00		
<1	0.852	0.457–1.592	0.616
1≤	0.684	0.203–2.309	0.541
Black coffee consumption (cups/day)			
Non-drink	1.00		
<1	1.361	0.548–3.381	0.507
1≤	1.718	0.707–4.175	0.233
Soft drink consumption (serving/day)			
Non-drink	1.00		
<1	1.186	0.644–2.186	0.584
1≤	6.326 **	1.908–20.971	0.003
Family medical history of Diabetes			
No	1.00		
Yes	2.077 *	1.141–3.784	0.017
Family medical history of Hypertension			
No	1.00		
Yes	1.495	0.832–2.685	0.179
Family medical history of Hyperlipidemia			
No	1.00		
Yes	1.618	0.912–2.870	0.100
Shift work			
Yes	1.00		
No	1.757 *	1.022–3.021	.041

* *p* < 0.05, ** *p* < 0.01.

## References

[1] Moore J.X., Chaudhary N., Akinyemiju T. (2017). Metabolic syndrome prevalence by race/ethnicity and sex in the United States: National health and nutrition examination survey, 1988–2012. Prev. Chronic Dis..

[2] Grundy S.M. (2016). Metabolic syndrome update. Trends Cardiovasc. Med..

[3] Palmer M.K., Toth P.P. (2019). Trends in lipids, obesity, metabolic syndrome, and diabetes mellitus in the United States: An NHANES analysis (2003–2004 to 2013–2014). Obesity.

[4] Li Y., Zhao L., Yu D., Wang Z., Ding G. (2018). Metabolic syndrome prevalence and its risk factors among adults in China: A nationally representative cross-sectional study. PLoS ONE.

[5] Park E., Choi S.J., Lee H.Y. (2013). The prevalence of metabolic syndrome and related risk factors based on the KNHANES V 2010. J. Agric. Med. Community Health.

[6] Ghosh A., Liu T., Khoury M.J., Valdez R. (2010). Family history of diabetes and prevalence of the metabolic syndrome in US adults without diabetes: 6-year results from the National Health and Nutrition Examination Survey (1999–2004). Public Health Genom..

[7] Lipińska A., Koczaj-Bremer M., Jankowski K., Kaźmierczak A., Ciurzyński M., Ou-Pokrzewińska A., Mikocka E., Lewandowski Z., Demkow U., Pruszczyk P. (2014). Does family history of metabolic syndrome affect the metabolic profile phenotype in young healthy individuals?. Diabetol. Metab. Syndr..

[8] Tucker J.M., Welk G.J., Beyler N.K., Kim Y. (2016). Associations between physical activity and metabolic syndrome: Comparison between self-report and accelerometry. Am. J. Health Promot..

[9] Hosseini Z., Whiting S.J., Vatanparast H. (2016). Current evidence on the association of the metabolic syndrome and dietary patterns in a global perspective. Nutr. Res. Rev..

[10] Chang H.E. (2017). The Effects of Job Demands and Job Resources on the Health and Well-Being of Hospital Nurse. Unpublished. Doctoral Dissertation.

[11] Kim K., Kim K., Park S.M. (2016). Association between the prevalence of metabolic syndrome and the level of coffee consumption among Korean women. PLoS ONE.

[12] Kim S.A., Shin S. (2019). The association between coffee consumption pattern and prevalence of metabolic syndrome in Korean adults. Nutrients.

[13] Suliga E., Kozieł D., Ciesla E., Rebak D., Głuszek-Osuch M., Głuszek S. (2019). Consumption of alcoholic beverages and the prevalence of metabolic syndrome and its components. Nutrients.

[14] Pietroiusti A., Neri A., Somma G., Coppeta L., Iavicoli I., Bergamaschi A., Magrini A. (2010). Incidence of metabolic syndrome among night-shift healthcare workers. Occup. Environ. Med..

[15] Ohkuma T., Hirakawa Y., Nakamura U., Kiyohara Y., Kitazono T., Ninomiya T. (2015). Association between eating rate and obesity: A systematic review and meta-analysis. Int. J. Obes..

[16] Tao L., Yang K., Huang F., Liu X., Li X., Luo Y., Wu L., Guo X. (2018). Association between self-reported eating speed and metabolic syndrome in a Beijing adult population: A cross-sectional study. BMC Public Health.

[17] Shin S., Kim S.A., Ha J., Lim K. (2018). Sugar-sweetened beverage consumption in relation to obesity and metabolic syndrome among Korean adults: A cross-sectional study from the 2012–2016 Korean National Health and Nutrition Examination Survey (KNHANES). Nutrients.

[18] Narain A., Kwok C.S., Mamas M.A. (2017). Soft drink intake and the risk of metabolic syndrome: A systematic review and meta-analysis. Int. J. Clin. Pract..

[19] Ley S.H., Ardisson Korat A.V., Sun Q., Tobias D.K., Zhang C., Qi L., Willett W.C., Manson J.E., Hu F.B. (2016). Contribution of the nurses’ health studies to uncovering risk factors for type 2 diabetes: Diet, lifestyle, biomarkers, and genetics. Am. J. Public Health.

[20] Ministry of Food and Drug Safety 2019 Food & Drug Statistical Yearbook (Publication No. 11-1471000-000165-10). https://www.mfds.go.kr/brd/m_371/view.do?seq=30718&srchFr=&srchTo=&srch.

[21] Pae S.J., Lim H.J., Kim J.Y., Kang H.T., Lee J.W. (2017). Health behavior and nutrient intake in metabolically abnormal overweight and metabolically abnormal obesity. Korean J. Health Promot..

[22] Marventano S., Salomone F., Godos J., Pluchinotta F., Rio D.D., Mistretta A., Grosso G. (2016). Coffee and tea consumption in relation with non-alcoholic fatty liver and metabolic syndrome: A systematic review and meta-analysis of observational studies. Clin. Nutr..

[23] Yeon J.Y., Bae Y.J. (2017). 3-in-1 coffee consumption is associated with metabolic factors in adults: Based on 2012~2015 Korea National Health and Nutrition Examination Survey. J. Nutr. Health.

[24] Je Y., Jeong S., Park T. (2014). Coffee consumption patterns in Korean adults: The Korean National Health and Nutrition Examination Survey (2001–2011). Asia Pac. J. Clin. Nutr..

[25] Kim S.K., Hong S.H., Chung J.H., Cho K.B. (2017). Association between alcohol consumption and metabolic syndrome in a community-based cohort of Korean adults. Med. Sc.i Monit..

[26] Hu X., Yu W., Yang L., Pan W., Si Q., Chen X., Li Q., Gu X. (2019). The association between first-degree family history of diabetes and metabolic syndrome. Endocr. Pract..

[27] Moon J.H., Roh E., Oh T.J., Kim K.M., Moon J.H., Lim S., Jang H.C., Choi S.H. (2017). Increased risk of metabolic disorders in healthy young adults with family history of diabetes: From the Korea National Health and Nutrition Survey. Diabetol. Metab. Syndr..

[28] Bello-Chavolla O.Y., Kuri-García A., Ríos-Ríos M., Vargas-Vázquez A., Cortés-Arroyo J.E., Tapia-González G., Gruz-Bautista I., Aguilar-Salinas C.A. (2018). Familial combined hyperlipidemia: Current nowledge, perspectives, and controversies. Rev. Investig. Clin..

[29] Skoumas I., Masoura C., Aznaouridis K., Metaxa V., Tsokanis A., Papadimitrious L., Tousoulis D., Pitsavos C., Stefanadis C. (2013). Impact of cardio-metabolic risk factors on major cardiovascular events in patients with familial combined hyperlipidemia. Circ. J..

[30] Ranasinghe P., Cooray D.N., Jayawardena R., Katulanda P. (2015). The influence of family history of hypertension on disease prevalence and associated metabolic risk factors among Sri Lankan adults. BMC Public Health.

[31] Canuto R., Garcez A.S., Olinto M.T. (2013). Metabolic syndrome and shift work: A systematic review. Sleep Med. Rev..

[32] Reiter R.J., Tan D.X., Korkmaz A., Ma S. (2012). Obesity and metabolic syndrome: Association with chronodisruption, sleep deprivation, and melatonin suppression. Ann. Med..

[33] Peplonska B., Bukowska A., Sobala W. (2015). Association of rotating night shift work with BMI and abdominal obesity among nurses and midwives. PLoS ONE.

[34] Khosravipour M., Shahmohammadi M., Athar H.V. (2019). The effects of rotating and extended night shift work on the prevalence of metabolic syndrome and its components. Diabetes Metab. Syndr..

[35] Terada T., Mistura M., Tulloch H., Pipe A., Reed J. (2019). Dietary behaviour is associated with cardiometabolic and psychological risk indicators in female hospital nurses—A post-hoc, cross-sectional study. Nutrients.

[36] Almajwal A.M. (2016). Stress, shift duty, and eating behavior among nurses in Central Saudi Arabia. Saudi Med. J..

[37] Kim O., Ahn Y., Lee H.Y., Jang H.J., Kim S., Lee J.E., Jung H., Cho E., Lim J.Y., Kim M.J. (2017). The Korea Nurses’ Health Study: A prospective cohort study. J. Women’s Health.

[38] Grundy S.M., Cleeman J.I., Daniels S.R., Donato K.A., Eckel R.H., Franklin B.A., Gordon D.J., Krauss R.M., Savage P.J., Smith S.C. (2005). Diagnosis and management of the metabolic syndrome: An American Heart Association/National Heart, Lung, and Blood Institute Scientific Statement. Circulation.

[39] Yen A.M.-F., Boucher B.J., Yueh-Hsia S., Fann J.C.-Y., Chen S.L.-S., Huang K.-C., Chen H.-H. (2016). Longer duration and earilier age of onset of paternal betel chewing and smoking increase metabolic syndrome risk in human offspring, independently, in a community-based screening program in Taiwan. Circulation.

[40] Kim D.-W., Her S.-H., Park H.W., Park M.-W., Chang K., Chung W.S., Seung K.B., Ahn T.H., Jeong M.H., Rha S.-W. (2019). Association between body mass index and 1-year outcome after acute myocardial infarction. PLoS ONE.

[41] Kim D.W., Song S., Lee J.E., Oh K., Shim J., Kweon S., Paik H.Y., Joung H. (2015). Reproducibility and validity of an FFQ developed for the Korea National Health and Nutrition Examination Survey (KNHANES). Public Health Nutr..

[42] Pan W., Bai H. (2016). Propensity score methods in nursing research: Take advantage of them but proceed with caution. Nurs. Res..

[43] Matilla-Santander N., Espinola M., Cartanyá-Hueso À., Lidón-Moyano C., González-Marrón A., Martín-Sánchez J.C., Cainzos-Achirica M., Sánchez J.M.M. (2020). Prevalence and determinants of metabolic syndrome in Spanish salaried workers: Evidence from 15,614 men and women. J. Public Health.

[44] Montano D. (2017). Association between socioeconomic determinants and the metabolic syndrome in the German Health Interview and Examination Survey for Adults (DEGS1)—A mediation analysis. Rev. Diabet. Stud..

[45] Kim M.H., Lee S.H., Shin K.S., Son D.Y., Kim S.H., Joe H., Woo B.W., Hong S.H., Sho C.Y., Shin H.S. (2020). The change of metabolic syndrome prevalence and its risk factors in Korean adults for decade: Korea National Health and Nutrition Examination Survey for 2008–2017. Korean J. Fam. Pract..

[46] Chico-Barba G., Jimenez-Limas K., Sanchez-Jimenez B., Samano R., Rodriguez-Ventura A.L., Castillo-Perez R., Tolentino M. (2019). Burnout and metabolic syndrome in female nurses: An observational study. Int. J. Environ. Res. Public Health.

[47] Bae Y., Choi S.-Y., Seo Y.-M. (2019). Factors affecting the metabolic syndrome of in adults aged the 20–30 years using the National Health and Nutrition Survey Data for 2016. Korean Data Anal. Soc..

[48] Yoshida J., Eguchi E., Nagaoka K., Ito T., Ogino K. (2018). Association of night eating habits with metabolic syndrome and its components: A longitudinal study. BMC Public Health.

[49] Bo S., Musso G., Beccuti G., Fadda M., Fedele D., Gambino R., Cassader M. (2014). Consuming more of daily caloric intake at dinner predisposes to obesity. A 6-year population-based prospective cohort study. PLoS ONE.

[50] Na S.-K., Cheon S.-H., Choi Y.-J., Lee H.-J., Roh Y.-K., Choi M.-K. (2016). Relationship between abdominal obesity and proportion of supper and late-night meals. Korean J. Obes..

[51] Madjd A., Taylor M.A., Delavari A., Malekzadeh R., Macdonald I.A., Farshchi H.R. (2016). Beneficial effect of high energy intake at lunch rather than dinner on weight loss in healthy obese women in a weight-loss program: A randomized clinical trial. Am. J. Clin. Nutr..

[52] Kutsuma A., Nakajima K., Suwa K. (2014). Potential association between breakfast skipping and concomitant late-night-dinner eating with metabolic syndrome and proteinuria in the Japanese population. Scientifica.

[53] Kregiel D. (2015). Health safety of soft drinks: Contents, containers, and microorganisms. Biomed. Res. Int..

[54] Funtikova A.N., Subirana I., Gomez S.F., Fito M., Elosua R., Benitez-Arciniega A.A., Schroder H. (2015). Soft drink consumption is positively associated with increased waist circumference and 10-year incidence of abdominal obesity in Spanish adults. J. Nutr..

[55] Lee H.S., Kwon S.O., Yon M., Kim D., Lee J.Y., Nam J., Park S.J., Yeon J.Y., Lee S.K., Lee H.Y. (2014). Dietary total sugar intake of Koreans: Based on the Korea National Health and Nutrition Examination Survey (KNHANES), 2008–2011. J. Nutr. Health.

[56] Dennis E.J., Kang M., Han S.N. (2017). Relation between beverage consumption pattern and metabolic syndrome among healthy Korean adults. Korean J. Community Nutr..

[57] Boyle M., Masson S., Anstee Q.M. (2018). The bidirectional impacts of alcohol consumption and the metabolic syndrome: Cofactors for progressive fatty liver disease. J. Hepatol..

[58] Anthanont P., Ramos P., Hames K.C. (2017). Family history of type 2 diabetes, abdominal adipocyte size and markers of the metabolic syndrome. Int. J. Obes..

[59] Yu K.H., Yi Y.H., Kim Y.J., Cho B.M., Lee S.Y., Lee J.G., Jeong D.W., Ji S.Y. (2017). Shift work is associated with metabolic syndrome in young female Korean workers. Korean J. Fam. Med..

[60] Nikpour M., Tirgar A., Hajiahmadi M., Hosseini A., Heidari B., Ghaffari F., Ebadi A., Nasiri F., Firouzbakht M. (2019). Shift work and metabolic syndrome: A multi-center cross-sectional study on females of reproductive age. Biomed. Rep..

[61] Loef B., van der Beek A.J., Holtermann A., Hulsegge G., van Baarle D., Proper K.I. (2018). Objectively measured physical activity of hospital shift workers. Scand. J. Work. Eenviron. Health.

